# Educational program for orthopedic surgeons’ influences for osteoporosis

**DOI:** 10.1515/med-2021-0365

**Published:** 2021-09-29

**Authors:** Huafeng Zhuang, Shufeng Lin, Yizhong Li, Siqing Cai, Peiwen Wang, Haiming Yu, Jinkuang Lin, Xuedong Yao, Hao Xu

**Affiliations:** Department of Orthopedics, The Second Affiliated Hospital of Fujian Medical University, No. 950 Donghai Street, Fengze District, Quanzhou, Fujian 362000, China; Department of Radiology, The Second Affiliated Hospital of Fujian Medical University, Quanzhou, Fujian 362000, China

**Keywords:** bone turnover markers, bone density, education, hip fracture, medication therapy management

## Abstract

**Background:**

In this retrospective study, we studied the impact of educational osteoporosis program on the rates of bone mineral density (BMD) assessment and bone turnover markers (BTM) and drug medications in the patients with hip fracture.

**Methods:**

This retrospective research enrolled 651 patients aged ≥50 years who experienced hip fractures from January 2013 to December 2015. We recorded whether patients had received BMD assessment, BTM measurement, and anti-osteoporosis therapy during the period of hospitalization. Orthopedic surgeons were classified into the trained group and the untrained group. The rates of BMD assessment, BTM measurement, and anti-osteoporosis medications in the patients with hip fracture were compared between the trained group and the untrained group.

**Results:**

BMD assessment was performed in 109/220 patients in the trained group and 142/431 patients in the untrained group. BTM measurements were performed in 130 patients in the trained group and 124 patients in the untrained group. Forty eight patients in the trained group and 63 patients in the untrained group received bisphosphonate medications.

**Conclusions:**

Although the rates of BMD assessment, BTM measurement, and bisphosphonate use in the patients after hip fractures are still insufficient, education programs help to improve the situation.

## 1 Introduction

Osteoporosis is very common in the elderly, and the consequences of fractures caused by osteoporosis are very serious. Hip fragility fractures are a common and serious complication of osteoporosis, which is associated with a high risk of fracture and mortality in the future [1,2]. The risk of hip fracture increases by 2.6-fold for each standard deviation decrease in Bone mineral density (BMD) of femoral neck and increases by 3.2-fold in the patients with previous hip fracture [3,4]. The prevention of new fracture for the patients is important after the surgical management of hip fragility fracture. BMD measured with dual-energy X-ray absorptiometry (DXA) is the gold standard of diagnosing osteoporosis. Several studies confirm that anti-osteoporosis medications can reduce the risk of future fractures and the mortality after hip fracture [5,6,7]. However, the rate of BMD assessment after hip fracture is low at 1.2–28.2% and the rate of osteoporosis medications is similar at 14.5% [8,9]. Recent study suggested that the rates of BMD assessment and anti-osteoporotic therapy after hip fracture increased 2-fold after active participation of orthopedic surgeons in the treatment of osteoporosis [10]. From 2013 to 2015, we performed an educational osteoporosis program each year. In order to see the influence of orthopedic surgeon’s awareness on the diagnosis and treatment of osteoporosis, this retrospective study compared the rates of BMD assessment, bone turnover markers (BTM), and drug medications in the patients with hip fracture between the trained orthopedic surgeons and untrained orthopedic surgeons in the same hospital.

## 2 Methods

### 2.1 Study population

This retrospective research enrolled 651 patients aged ≥50 years who experienced hip fragility fractures. The patients were admitted to the Second Affiliated Hospital of Fujian Medical University in China from January 2013 to December 2015. The inclusion criteria in our study were the patients admitted with new hip fracture, low-energy trauma such as a fall, and patients’ aged ≥50 years. Patients with pathological fractures caused by malignant tumors, fracture due to high-energy trauma, failure of prosthesis, and those aged below 50 years old were excluded. The patients’ medical records were retrospectively reviewed. We recorded whether patients had received BMD measurement with DXA, measurement of bone turnover markers including serum procollagen type I N propeptide (s-PINP) and serum Cterminal cross-linking telopeptide of type I collagen (s-CTX), and anti-osteoporosis therapy during the period of hospitalization. Anti-osteoporosis drug included bisphosphonates (zoledronate, alendronate, risedronate, ibandronate) with supplementation of calcium and vitamin D, non-bisphosphonates (calcitonin, active vitamin D analogues, and raloxifene) with supplementation of calcium and vitamin D, and basic supplementation of calcium and vitamin D.

An educational osteoporosis program was held each year from 2013 to 2015. The education osteoporosis program provided the information regarding the guidelines of diagnosis and treatment for osteoporosis, the association between osteoporosis and hip fracture, DXA for diagnosis of osteoporosis, clinical application of bone turnover markers, anti-osteoporotic drugs for prevention of fractures, the importance of drug compliance for osteoporosis, etc. A group of six orthopedic surgeons who worked in the same medical team participated in the educational program of osteoporosis each year and completed the educational programs. There were 36 orthopedic surgeons at the second affiliated hospital of Fujian Medical University between 2013 and 2015. Orthopedic surgeons were classified into the trained group and the untrained group on the basis of whether or not to participate in the educational osteoporosis programs. The orthopedic surgeons independently decided the management of osteoporosis in the patients with hip fracture. The rates of BMD assessment, BTM measurement, and anti-osteoporosis medications were compared between the trained group and the untrained group.

**Ethics approval and consent to participate:** All participants were informed at the time of admission to hospital that their data would be included in a research study and provided written consent. Approval for this research was given by the ethics committee of the Second Affiliated Hospital of Fujian Medical University (2016-91).**Consent for publication:** Not applicable.

### 2.2 Statistical analysis

All statistical analyses were performed using SPSS statistical software (SPSS, version19.0; SPSS Inc, Chicago, Illinois). Measurement data were expressed as mean ± standard deviation. All parameters of groups were compared with chi square test. *p* < 0.05 was considered as statistically significant.

## 3 Results

There were 651 patients enrolled in this retrospective research. Patients’ age was from 50 to 103, with the average of 78.02 ± 10.09 years. There were 432 females and 219 males. There were 324 patients with fractures of femoral neck and 327 patients with intertrochanteric fractures of femur. The internal fixation was performed for 299 patients, hip arthroplasty for 270 patients, and nonoperative treatment for 82 patients.

Among 651 patients with hip fractures, there were 220 patients in the trained group of orthopedic surgeons and 431 patients in the untrained group.

### 3.1 BMD assessment

BMD assessment was performed in 251 of 651(38.6%) patients including 109 patients in the trained group and 142 patients in the untrained group. The rate of BMD assessment in the trained group was significantly higher than that in the untrained group (Table 1).There were 198 patients with BMD *T*-score ≤−2.5 at femoral neck or lumbar spine and 53 cases with BMD *T*-score >−2.5 at femoral neck and/or lumbar spine in BMD assessment.

**Table 1:** Comparison of rate of BTMs and BMD between the trained group and untrained group [*n*, (%)]

	Trained group	Untrained group	*χ*²	*p*
Patients	220	431		
BTM test	130 (59.0%)	124 (28.8%)	56.277	<0.005
BMD test	109 (49.5%)	142 (32.9%)	16.940	<0.005

### 3.2 BTM measurement

BTM measurement including serum procollagen type I N propeptide (s-PINP) and serum Cterminal cross-linking telopeptide of type I collagen (s-CTX) was performed in 254 of 651 (39.0%) patients including 130 patients in the trained group and 124 patients in the untrained group. The rate of BTM measurement in the trained group was significantly higher than that in the untrained group (Table 1).

### 3.3 Anti-osteoporosis medications

There was no anti-osteoporosis medication for 84 (12.9%) patients. The basic supplementation of calcium and vitamin D was given to 77 (11.8%) patients, non-bisphosphonate with supplementation of calcium and vitamin D was given to 379 (58.2%) patients, and bisphosphonate with supplementation of calcium and vitamin D was given to 111 (17.1%) patients. Calcitonin, calcitriol, alfacalcidol, and zoledronate were often used drugs and zoledronate was only used as one of bisphosphonates in our patients. The rate of zoledronate use in the trained group was significantly higher than that in the untrained group (*p* < 0.05) (Table 2).

**Table 2:** Comparison of drug therapies for osteoporosis between the trained group and untrained group [*n*, (%)]

	Trained group	Untrained group	*χ*²	*p*
Patients	220	431		
No medication	28 (12.7%)	56 (13.0%)	0.009	>0.05
Calcium + D	15 (6.8%)	62 (14.4%)	7.997	<0.05
Bisphosphonates	48 (21.8%)	63 (14.6%)	4.721	<0.05
Non-bisphosphonates	129 (58.6%)	250 (58.0%)	0.024	>0.05

## 4 Discussion

This retrospective study confirmed that the rates of BMD assessment and BTM measurement were 38.6 and 39.0%, respectively, in the patients with hip fracture. The patients in the trained group of orthopedic surgeons had higher rates of BMD assessment and BTM measurement than the patients in the untrained group (49.5% vs 32.9% for BMD assessment and 59.0% vs 28.8% for BTM measurement). This study also confirmed that non-bisphosphonate medications were administered to half of patients by orthopedic surgeons and bisphosphonate medication was only administered to 17.1% of the patients with hip fracture. More bisphosphonate was administered to patients in the trained group than in the untrained group (21.8% vs 14.6%).

BMD assessment is indicated for the patients with fragility fracture. The result of BMD assessment is necessary for orthopedic surgeons to know the severity of bone loss, to assess the risk of new fracture, to make the decision of starting anti-osteoporosis therapy, and to evaluate the effect of drug therapy in the future. The result of BMD assessment also is a good basis of communication between surgeons and patients. However, the low rate of BMD assessment after fragility fractures is popular in the world. In 2005, Vanasse et al. [11] reported that only 4.6% men and 13.1% women received BMD testing after a fragility fracture in Canada.

Shibli-Rahhal et al. [8] reported that only 1.2% of hip fracture patients underwent BMD testing in U.S. Department of Veterans Affairs hospitals between 2004 and 2006. Kung et al. [9] reported in 2013 that only 28.2% of patients after a fragility hip fracture had BMD measurement in Asia. Nguyen et al. [12] reported in 2018 that 32% patients had DXA scan after hip fracture. It seems to be a rising trend of BMD assessment in recent years. In this study, the rate of BMD assessment was 38.6%. The rate of BMD assessment significantly increased from 33.2% patients in the untrained group to 48.6% patients in the trained group. It is confirmed that BMD assessment can significantly increase the initiation of bisphosphonate therapies in the patients with hip fracture during the period of hospitalization [13].

BTM measurement has allowed estimation of bone turnover state, prediction of the rate of BMD change in near future, assessment of the effect of drug treatment, evaluation of bone quality, and prediction of the risk of fractures [14]. International Osteoporosis Foundation/International Federation of Clinical Chemistry and Laboratory Medicine recommends s-PINP and s-CTX to be used as reference markers in observational and intervention studies [15]. In this study, 39.0% patients with hip fracture had BTM measurement including s-PINP and s-CTX and the rate of BTM measurement significantly increased from 28.8% patients in the untrained group to 59% patients in the trained group. The increased rates of BMD assessment and BTM measurement in the trained group suggested that educational program had strong impact on orthopedic surgeons’ application of BMD assessment and BTM measurement for patients with hip fracture.

Anti-osteoporosis medications are indicated in the patients with hip fragility fracture for prevention of future fracture because a prior fracture significantly increases future fracture risk. 10, 18, and 31% of women aged ≥65 years fractured again within 1, 2, and 5 years, respectively, following their initial clinical fracture and the risk of hip fracture within 1, 2, and 5 years following any clinical fracture was 2.4, 4.8, and 10.2%, respectively [16]. Several drugs showed to significantly reduce the future fracture in the patients with fragility fracture. Zoledronate reduced new clinical vertebral fracture (RRR 46%) in the patients with hip fracture. Denosumab significantly reduced the incidence of any subsequent fracture (RRR 39%). Teriparatide reduced new vertebral fractures (RRR 65%) and non-vertebral fragility fractures (RRR 53%) in postmenopausal women with vertebral fractures [5]. However, the treatment gap of osteoporosis is evident throughout the world. Wilk et al. [17] reported in 2014 that only 18% received osteoporosis therapy within 90 days and 23% within 1 year postfracture in USA. Keshishian et al. [18] reported in 2017 that only 27.7% were treated with osteoporosis therapy within 12 months of index fracture and 72.2% were untreated. Kim et al. [19] reported that the initiation rate of osteoporosis treatment following hip fracture was 23.1% in Korea. In this study, 50.5% patients received non-bisphosphonate medication and only 17.1% patients received bisphosphonate medication. The reasons for many patients taking the non-bispho sphonate medication are discussed in the following. First, the non-bisphosphonate therapies such as calcitonin, calcitriol, and alfacalcidol are recommended for patients with osteoporosis by Chinese guideline [20]. Second, oral bisphosphonates have strict and tedious administration requirements which is inconvenient for elderly patients with hip fracture and high incidence of adverse events such as headache, musculoskeletal pain, and fever after intravenous infusion of zoledronate leads to patients’ worry for drug safety. Third, the use of anti-osteoporosis drugs was also influenced by patients’ perception of bone health, policy of controlling medical expense, and medical insurance payment. Bisphosphonates are first-line drugs for osteoporosis and have strong evidence and good results of reducing fracture risk.

**Figure 1:**
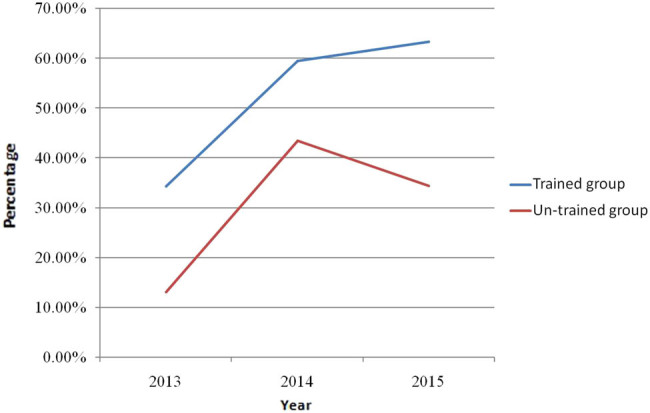
The rate of bone mineral density test in the patients with hip fracture in trained group and untrained group between 2013 and 2015.

**Figure 2:**
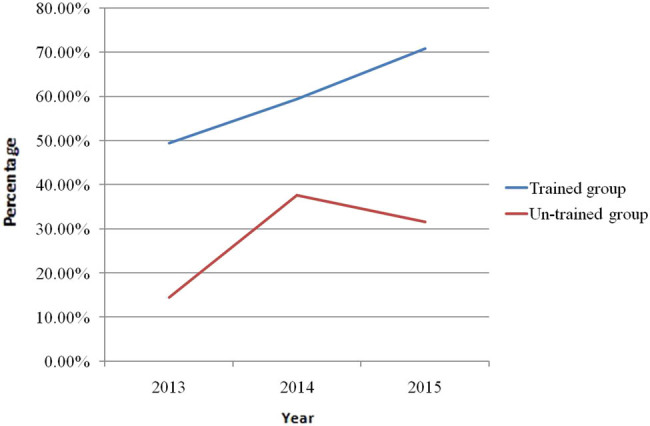
The rate of bone turnover marker test in the patients with hip fracture in trained group and untrained group between 2013 and 2015.

**Figure 3:**
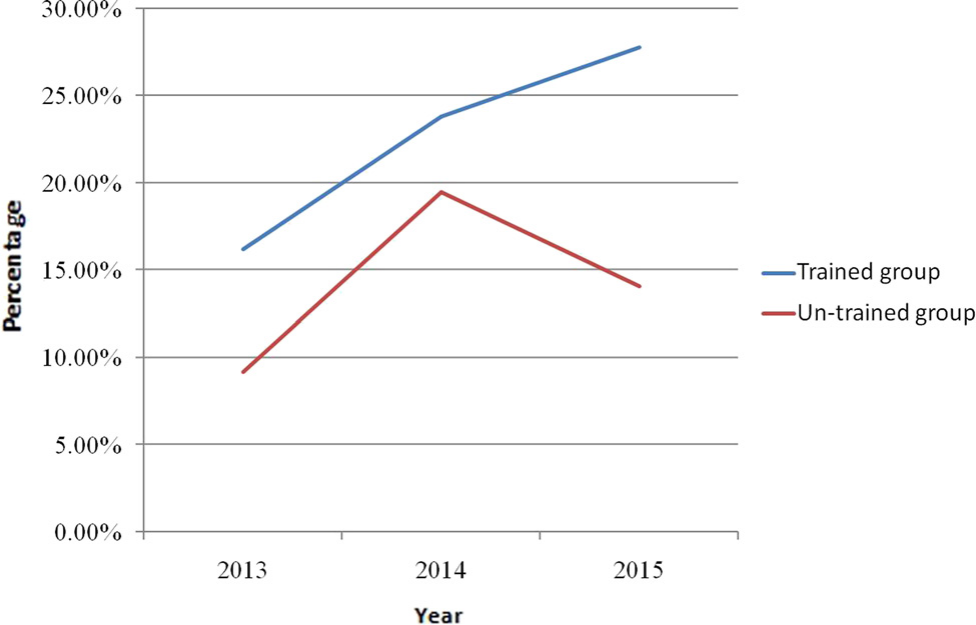
The rate of bisphosphonate use in the patients with hip fracture in trained group and untrained group between 2013 and 2015.

The care gap of osteoporosis is evident throughout the world. Low diagnosis and treatment rate of osteo porosis after fragility fracture suggests that the orthopedic surgeons fail to adhere to the guidelines for osteoporosis. In a survey of 2,910 orthopedic surgeons, the majority of orthopedic surgeons lacked knowledge of osteoporosis and sufficient training in management of patients with osteoporosis [21]. AO Spine Latin America Survey of 349 spine surgeons suggested that only 19.6% of respondents practiced screening for osteoporosis prior to surgery [22]. In a survey of orthopedic surgeons’ views on the osteoporosis care gap, they recognized the importance of osteoporosis care and the existence of a care gap whereby patients with fragility fractures were often not evaluated or treated for osteoporosis and supported an increasing role for orthopedic surgeons in screening for osteoporosis, but many expressed reservations about taking responsibility for initiating osteoporosis treatment [23]. A cross-sectional survey of 452 surgeons in China showed that the sensitivity of orthopedic surgeons to the prevention of secondary fractures was relatively low and that the continuing medical education was required to encourage surgeons to take greater responsibility for screening, treating, educating, and following their patients with fragility fractures [24]. Nelson et al. [25] confirmed that the residents’ participation in the musculoskeletal training program was associated with significant improvements in their completion of DXA scans, diagnosis of osteoporosis, and initiation of fracture-reducing medications. Osteoporosis was paid more and more attention by orthopedic surgeons. Our study showed that there was an increasing tendency in the rates of BMD assessment, BTM measurement, and bisphosphonate use in the patients in the untrained group from 2013 to 2015, which reached its peak in 2014 and went down in 2015. However, the rates of BMD assessment, BTM measurement, and bisphosphonate use in the trained group increased steadily from 2013 to 2015 and were highest in 2015 (Figures 1–3). Our study confirmed that educational program improved surgeons’ management of osteoporosis. Osteoporosis is associated with multiple disciplines and educational program is important and an effective measure for improvement of physicians’ and surgeons’ knowledge of osteoporosis.

Several limitations of this study should be considered. The type of medical insurance for these patients was different and an average payment of medical costs to hospitals for hip fracture covered by insurance also was different. We did not assess orthopedic surgeons’ and patients’ knowledge of osteoporosis which might affect the rate of osteoporosis diagnosis and treatment after a hip fracture.

## Abbreviations

BMD: bone mineral density
DXA: dual-energy X-ray absorptiometry
BTM: bone turnover markers
s-PINP: serum procollagen type I N propeptide
s-CTX: serum Cterminal cross-linking telopeptide of type I collagen
